# Assessment of Psoas Muscle Index in Middle-Aged Type 2 Diabetes Patients: Impact of Insulin Therapy on Sarcopenia

**DOI:** 10.3390/tomography10070079

**Published:** 2024-07-10

**Authors:** Ismail Taskent, Bunyamin Ece, Sonay Aydin

**Affiliations:** 1 Department of Radiology, Kastamonu University, 37150 Kastamonu, Turkey; bunyaminece@kastamonu.edu.tr; 2 Department of Radiology, Erzincan University, 24100 Erzincan, Turkey; sonay.aydin@erzincan.edu.tr

**Keywords:** sarcopenia, type 2 diabetes mellitus, psoas muscle index, insulin, psoas muscle density, computed tomography

## Abstract

Objective: Sarcopenia, characterized by progressive skeletal muscle loss, poses significant health risks, including physical impairment and mortality. The relationship between sarcopenia and insulin resistance suggests insulin therapy’s potential in preserving muscle mass, particularly in Type 2 diabetes mellitus (T2DM) patients. This study aims to evaluate the Psoas Muscle Index (PMI) via computed tomography (CT) in middle-aged T2DM patients on insulin therapy versus oral antidiabetic drugs (OAD) and controls. Methods: This retrospective study included 107 middle-aged T2DM patients undergoing non-contrast CT scans and 58 age-matched controls. CT images were analyzed to calculate PMI. Statistical analysis included Chi-square tests, independent samples *t*-tests, Mann–Whitney U tests, and correlation analyses. Results: Insulin-treated patients exhibited higher PMI than OAD users (*p* < 0.001), while OAD users had lower PMI than controls (*p* < 0.001). No significant difference was found between insulin-treated patients and controls (*p* = 0.616). Negative correlations were observed between T2DM duration/age and PMI across all groups, with a positive correlation between T2DM duration and BMI observed in the OAD group. Conclusions: Insulin therapy in T2DM patients, regardless of age or disease duration, positively impacts muscle mass, highlighting its potential in preserving muscular health and advocating for tailored treatment strategies in T2DM management.

## 1. Introduction

Type 2 diabetes mellitus (T2DM) is characterized by glucose intolerance, insulin resistance, inflammation, advanced glycation end-product buildup, and elevated oxidative stress. These characteristics can have a detrimental impact on a variety of elements of muscle health, and as a result, they considerably contribute to the worsening of sarcopenia [1,2,3,4]. The prevalence of sarcopenia is notably higher in T2DM patients, with estimates suggesting a bidirectional relationship between these conditions [5].

Sarcopenia is a syndrome defined by a progressive and widespread loss of skeletal muscle mass and strength, with the potential for negative effects such as physical impairment, poor quality of life, and mortality [6,7,8]. Early detection and intervention are crucial as sarcopenia is a treatable condition with appropriate musculoskeletal therapy [9].

Irwin Rosenberg [10] defined sarcopenia for the first time in 1988 as an age-related decline in skeletal muscle mass and function. In 1998, Baumgartner et al. [11] developed a method for assessing sarcopenia based on appendicular lean mass adjusted for height (kg/m^2^). While muscle mass and quality have been assessed using a variety of procedures and technologies in the past, computed tomography (CT) measurements are now considered the gold standard in studies [12,13,14,15].

The first preferred approach for determining total body muscle mass is the calculation of the psoas muscle index from lumbar 3rd vertebral (L3) level measurements. According to studies, the muscle mass acquired from a single section can provide an estimate of the whole body’s muscle mass [12,16]. Studies indicate that the psoas muscle index (PMI), calculated by dividing the cross-sectional area of the right and the left psoas at the L3 level by the square of the height, correlates positively with the total skeletal muscle volume and is effective for the diagnosis of sarcopenia [17,18,19].

Recent studies have highlighted several mechanisms by which T2DM exacerbates sarcopenia. Hyperglycemia-induced oxidative stress and chronic inflammation are primary contributors to muscle degradation in diabetic patients [20,21]. Additionally, advanced glycation end products (AGEs) accumulate in muscle tissue, further impairing muscle function and regeneration [21].

The relationship between sarcopenia and insulin resistance is well documented. Insulin resistance exacerbates muscle protein degradation and reduces muscle protein synthesis, contributing to muscle wasting in T2DM patients [22,23]. Although prior research has demonstrated that insulin sensitizers can increase skeletal muscle mass by inhibiting protein breakdown, the effect of insulin treatment on skeletal muscle mass remains unclear [24,25,26].

Sugimoto et al. [27] discovered that improving glycemic control and using insulin were significantly associated with increases in skeletal muscle mass in T2DM patients. Tanaka et al. [28] found that a reduction in endogenous insulin is an independent risk factor for diabetes-related sarcopenia. In another study, Bouchi et al. [23] discovered that patients with type 2 diabetes treated with insulin may be at a lower risk for the loss of skeletal muscle mass in the lower extremities compared with those who do not receive insulin treatment. It should be noted that the majority of the patients and control subjects in these reports were elderly.

Whereas previous research has focused on elderly patients, the aim of this retrospective study was to evaluate the CT measurements of the PMI, which is a radiological finding of sarcopenia in middle-aged patients with T2DM, and compare them with those of the normal population.

## 2. Materials and Methods

### 2.1. Participants

The study included 107 patients with Type 2 Diabetes Mellitus (T2DM) aged 35–65 years and 58 age-matched controls. Among the T2DM patients, 55 were treated with Oral Antidiabetic (OAD) medications and 52 with insulin. The inclusion criteria comprised patients aged 35–65 years, diagnosed with T2DM at least 3 years prior. Individuals with organ failure (*n* = 17), users of any drug that alters body composition (*n* = 3), individuals with a body mass index (BMI) greater than 35 (*n* = 12) or less than 18 (*n* = 3) kg/m^2^, and users of illegal drugs or hormonal or nutritional supplements (*n* = 4) were excluded from the study. Patients with psoas asymmetry (*n* = 6) due to scoliosis or for other reasons were also excluded. This process ensured that the final sample size remained at 107 patients.

In this study, 107 patients who presented to our hospital with complaints such as abdominal pain, kidney stones, or other conditions requiring a non-contrast abdominal CT scan were included. These CT scans were performed for various reasons, providing a diverse patient sample.

For the control group, 58 patients with comparable age and gender distribution were selected. All participants were investigated retrospectively by thoroughly examining hospital records. This included reviewing detailed laboratory results and ensuring that the control subjects were not diagnosed with diabetes mellitus nor receiving any diabetes treatment.

The data used for this study were collected anonymously, and local ethics committee approval was obtained for this study (ethics committee number: E-145678230-2347510.003). This study adhered to the Declaration of Helsinki. Because the study was retrospective, informed consent was not obtained.

### 2.2. Psoas Muscle Assessment

Abdominal CT scans of the 107 patients and 58 participants in the control group were evaluated. Non-contrast abdominal CT examinations were performed using a 64-detector CT scanner (Revolution EVO; GE Medical Systems, Chicago, IL, USA) in our clinic for any reason were used for analysis. The CT scan parameters comprise a tube potential of 120 kV, a beam collimation of 0.625 mm, mA ranging from 140 to 250, a rotation time of 0.5 s, and a reconstructed section thickness of 3 mm.

In the psoas muscle measurement process, the L3 level was determined to be the benchmark. The images were evaluated using CT images, which were transferred to an Advantage Windows workstation (ADW 4.7 Ext. 16 Software, GE Medical Systems, Chicago, IL, USA), and the psoas muscle areas were assessed using workstation’s software.

**Figure 1:**
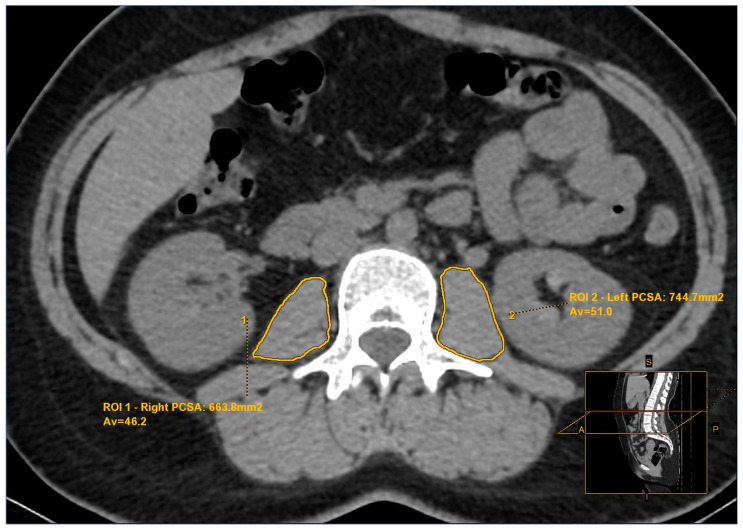
Psoas Muscle Assessment. The outer edge of the major psoas muscle was traced manually to assess the psoas cross-sectional area (PCSA) (mm^2^) and average (Av) attenuation values in Hounsfield Units (HU) at the lumbar third vertebral level with the free hand region of interest (ROI). ROI1: Right psoas muscle measurements. ROI2: Left psoas muscle measurements.

The free hand region of interest (ROI) was traced manually to assess the psoas cross-sectional area (PCSA) and attenuation values in Hounsfield Units (HU) of the outer edge of the psoas muscles at the L3 level. The PMI was calculated by evaluating all images, regardless of other clinical parameters or patient outcomes. To calculate the PMI (mm^2^/m^2^), the sum of the left and right PCSA (cm^2^) was divided by the square of the height (m^2^). Bilateral psoas muscle density (PMD) was expressed in Hounsfield Units (HU) by calculating the mean psoas muscle attenuation value (Figure 1).

### 2.3. Statistical Analysis

Data were analyzed using the Statistical Package for Social Sciences (SPSS) for Windows version 20 software (IBM SPSS Inc., Chicago, IL, USA). The conformity of the data to normal distribution was assessed by the Kolmogorov–Smirnov test. Numerical variables with a normal distribution are shown as the mean ± standard deviation (SD) values, variables without a normal distribution as median (minimum–maximum) values, and categorical variables as number (n) and percentage (%). A Chi-square test was used to compare categorical variables. For comparison between the groups, the independent samples *t* test was used for data with normal distribution, and the Mann–Whitney U test was used for data without normal distribution. Pearson correlation analysis was used for data with normal distribution, and Spearman correlation analysis was used for data without normal distribution. A reliability analysis test was applied to determine the interrater reliability levels between two different physicians, and ICC (Intraclass correlation coefficient) was calculated. ICC values from 0.75 to 1.0 were considered excellent, and ICC values from 0.40 to 0.75 were considered quite good [29]. A value of *p* < 0.05 was regarded as statistically significant.

## 3. Results

The participants had a mean age of 49.4 ± 9.1 years, with 46.1% (*n* = 76) being male and 53.9% (*n* = 89) female. Insulin-treated patients had a significantly higher Psoas Muscle Index (PMI) (618 ± 95 mm^2^/m^2^) compared to OAD users (533 ± 85 mm^2^/m^2^, *p* < 0.001). Additionally, the PMI of OAD users was significantly lower than that of the control group (627 ± 103 mm^2^/m^2^, *p* < 0.001), while no significant difference was found between insulin-treated patients and the control group (*p* = 0.616). Furthermore, the total Psoas Cross-Sectional Area (PCSA) was higher in the insulin group (1579 ± 278 mm^2^) compared to the OAD group (1459 ± 225 mm^2^, *p* = 0.015) and lower in the OAD group compared to the control group (1613 ± 307 mm^2^, *p* = 0.003). There were no significant differences in Psoas Muscle Density (PMD) across all groups.

The age and gender distribution displayed no statistically significant difference between the study and control groups, both in the overall population (healthy + patients) and when each group was assessed independently (*p* > 0.05) (Table 1).

Detailed comparisons of Body Mass Index (BMI), total Psoas Cross-Sectional Area (PCSA), Psoas Muscle Index (PMI), Psoas Muscle Density (PMD), the duration of Type 2 Diabetes Mellitus (T2DM), and HbA1c measurement results are presented in Table 2.

**Figure 2:**
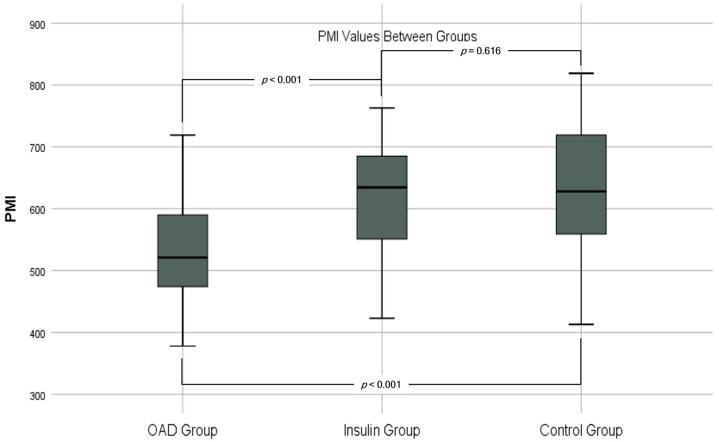
Graph of psoas muscle index (PMI) values and *p* values.

In group comparisons, PMI values were significantly higher in the insulin group compared to the OAD group (*p* < 0.001). The OAD group exhibited significantly lower PMI values than the control group (*p* < 0.001), while no significant difference was observed between the insulin group and the control group (*p* = 0.616) (Table 2) (Figure 2).

No significant difference was found in total PCSA measurements between the insulin group and the control group (*p* = 0.544). However, the OAD group presented with a significantly lower total PCSA compared to both the insulin group and the control group (*p* = 0.015 and *p* = 0.003, respectively) (Table 2).

BMI values were only significantly higher in the insulin group compared to the control group (*p* = 0.036), with no significant differences between the insulin group and the OAD group or between the OAD group and the control group (*p* > 0.05) (Table 2).

The duration of Type 2 DM was significantly higher in the insulin group compared to the OAD group (*p* < 0.001). No significant differences were observed between any group in density or HbA1c values (*p* > 0.05) (Table 2). Inter-rater reliability values (ICC) in PCSA measurements were 0.838 (95% CI, 0.730–0.903), indicating an excellent correlation.

**Figure 3:**
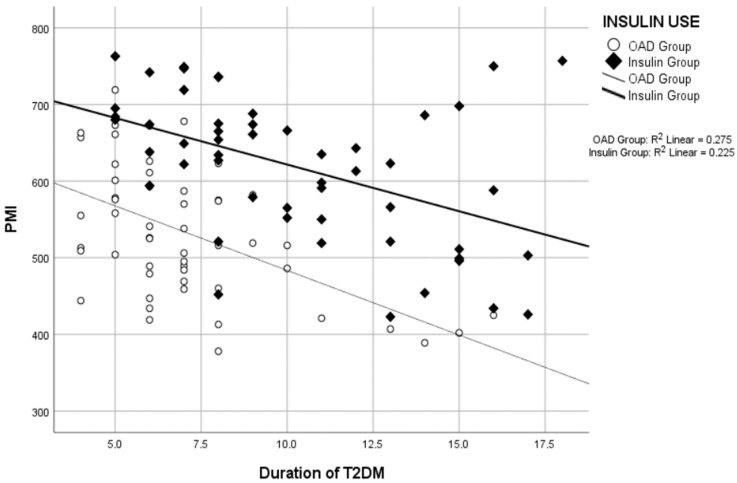
Correlation analysis graph between the duration of Type 2 diabetes mellitus (T2DM) and psoas muscle index (PMI).

**Figure 4:**
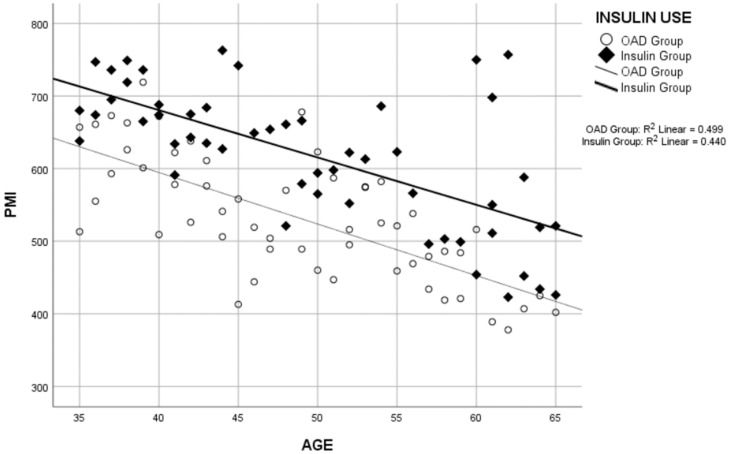
Correlation analysis graph between the age and psoas muscle index (PMI).

Correlation analyses revealed a negative correlation between the duration of T2DM and PMI (Figure 3) and between age and PMI (Figure 4) across the total study group, OAD group, and insulin group separately. While no correlation was found between the duration of Type 2 DM and BMI in the insulin group, a significant positive correlation was observed in the OAD group and the total study group (Table 3).

## 4. Discussion

Our study revealed a significant decrease in Psoas Muscle Index (PMI) values among middle-aged Type 2 Diabetes Mellitus (T2DM) patients compared to the control group, while no significant difference was observed between the insulin-using group and the control group. These findings suggest that middle-aged T2DM patients treated with insulin may have a reduced risk of sarcopenia compared to those treated solely with oral antidiabetic medications (Table 2).

Although the mechanism of sarcopenia in T2DM remains unclear, decreased endogenous insulin secretion has been implicated in reducing muscle mass in diabetic patients [23]. Insulin resistance and sarcopenia share a close association, as insulin plays a crucial role in maintaining muscle protein synthesis and degradation balance [30]. The cytoprotective effect of oral antidiabetic medications in T2DM patients remains uncertain [31]. Recent studies have shown accelerated declines in muscle function and mass in individuals with T2DM, with insulin therapy associated with muscle mass preservation [32]. Insulin’s anabolic properties promote muscle protein synthesis and inhibit breakdown, potentially aiding both T2DM treatment and muscle dysfunction management. However, while insulin therapy increases protein anabolism in younger T2DM patients, it may not confer beneficial effects regarding muscle hypertrophy or function in older individuals [31,33]. Consistent with this, recent research indicates significant declines in muscle mass and strength among elderly T2DM patients compared to normoglycemic controls [2,34]. Aging’s role in these changes is often indistinguishable from disease [32,34,35]. Hence, we excluded T2DM patients over 65 years old from our study to mitigate age-related sarcopenia effects.

The findings of our study are consistent with Shishikura et al.’s study [36], which showed a negative correlation between skeletal muscle mass and endogenous insulin secretion in T2DM patients and emphasized the critical role of insulin therapy in maintaining muscle mass. Similarly, Kim et al.’s [37] results highlighting the increased risk of sarcopenia in diabetic patients with normal BMI reinforce our observation of a limited correlation between BMI and PMI values.

While various methods and technologies have been used in the past to evaluate muscle mass and quality, studies now regard CT measurements as the most reliable and accurate approach. This is due to CT’s exceptional accuracy in distinguishing between different tissue types, such as muscle and fat, and its capacity to provide highly detailed cross-sectional images. CT imaging allows for the precise measurement of muscle area, density, and quality, which is crucial for diagnosing conditions like sarcopenia. The reliability of this method, compared to other techniques like dual-energy X-ray absorptiometry (DXA) and magnetic resonance imaging (MRI), makes it a preferred choice. Additionally, CT scans can be seamlessly integrated into clinical workflows, providing quick and easy assessments [12,13,14,15].

As fat has a high negative attenuation value for CT imaging, decreased muscle attenuation primarily reflects an increased fat content around muscle fibers [38]. Thus, we evaluated muscle mass and quality separately by assessing Psoas Muscle Density (PMD) in addition to PMI assessments (Figure 1). Non-contrast abdominal CT scans of patients in both groups were analyzed to minimize differences in CT acquisition parameters and image analysis techniques. No significant difference in PMD values was observed between the study and control groups, indicating that the PMI disparity directly relates to muscle mass.

Uncontrolled hyperglycemia leads to catabolism, potentially resulting in muscle protein breakdown and inadequate energy utilization, contributing to poor muscular performance. Poor glycemic control in diabetes is associated with increased systemic inflammatory cytokines, which negatively impacts muscle performance [35]. Studies have found associations between poor glycemic management, high HbA1c levels, and muscular weakness and sarcopenia [39,40]. However, in our study, no association was found between HbA1c levels and PMI values. This discrepancy may stem from HbA1c levels reflecting short-term glycemic management, while CT measurements and HbA1c values were obtained at different times.

Our findings revealed no statistically significant correlation between BMI and PMI (Table 3). T2DM patients with a high body fat percentage and low BMI face an increased sarcopenia risk. However, sarcopenia risk assessment in diabetic patients should not solely rely on BMI. While a low BMI and high body fat percentage increase the likelihood of sarcopenia, insulin therapy’s muscle-mass-loss-inhibitory effects may be BMI-independent [23,41].

A longer T2DM duration increases sarcopenia risk among patients [28,42]. We observed a significant negative correlation between T2DM duration and PMI, with insulin-using patients having a much longer diabetes duration than those using oral antidiabetic medications. Despite this, PMI values were significantly higher in the insulin group than the oral antidiabetic group (Table 2 and Table 3 and Figure 2 and Figure 3), indicating that insulin therapy reduces sarcopenia risk regardless of diabetes duration.

Research has shown that age-related changes significantly affect muscle quality and quantity. In a study by Prashanthi et al., standardized PMI measurements were made using CT and it was found that average PMI values decreased significantly with age in the Indian population [43]. Similarly, Zannoni et al. emphasized that the attenuation values of the psoas muscles correlate well with other hip muscles and that age-related muscle quality changes can be reliably evaluated with CT scans in different anatomical regions [44]. This reinforces the findings of our study that PMI can serve as a robust indicator of muscle health in T2DM patients.

A notable aspect of our study is its retrospective evaluation of T2DM patients under long-term treatment. While the literature predominantly reports findings from elderly subjects, our study sheds light on early sarcopenia diagnosis in middle-aged T2DM patients. Our results confirm that insulin therapy prevents early muscle mass reduction in T2DM patients, irrespective of disease duration, metabolic control, or age.

Our study has several limitations:

1. The retrospective design precluded obtaining data on muscle strength and function, which are essential for a comprehensive definition of sarcopenia.
2. Due to the retrospective nature of the study, we were unable to analyze the short- and long-term effects of patient medications.
3. The small sample size limits the generalizability of our findings.
4. We did not have sufficient information on the physical activity levels of the patients.

Further research is needed to validate our findings and explore potential contributing factors.

## 5. Conclusions

Our study underscores the significance of insulin therapy in mitigating sarcopenia risk among Type 2 Diabetes Mellitus (T2DM) patients. The findings highlight the critical role of insulin in preserving muscle mass and quality, irrespective of diabetes duration, metabolic control, or age. This suggests a promising avenue for managing sarcopenia in T2DM patients.

Moving forward, further research is warranted to validate these findings and explore potential mechanisms underlying the protective effects of insulin therapy. Additionally, future studies should investigate the long-term impacts of different antidiabetic medications on muscle health and function in T2DM patients. Such investigations can inform tailored treatment approaches aimed at optimizing both glycemic control and muscular well-being in this population.

## Figures and Tables

**Table 1:** Age and gender distribution of the participants.

	Study Group			Control Group ^d^ (*n* = 58)	^a,b^ *p* Value	^c,d^ *p* Value
	Insulin ^a^ (*n* = 52)	OAD ^b^ (*n* = 55)	Total Study Group ^c^ (*n* = 107)			
Age * (year) (Mean ± SD)	49.6 ± 9.6	48.7 ± 8.5	49.1 ± 9.0	50.0 ± 9.3	0.592	0.576
Gender ** (Male/Female), *n* (%)	24(46)/ 28(54)	25(45)/ 30(55)	49(46)/ 58(54)	27(47)/ 31(53)	0.548	0.528

* Independent samples *t* test ** Chi-square test, SD: standard deviation, OAD: oral antidiabetics. Superscript letters were added so that *p* values could be compared in pairs.

**Table 2:** Data in the study and control group and comparison between groups.

	Study Group			Control Group ^d^ (*n* = 58)	^a,b^ *p* Value	^a–d^ *p* Value	^b–d^ *p* Value	^c,d^ *p* Value
	Insulin ^a^ (*n* = 52)	OAD ^b^ (*n* = 55)	Total Study Group ^c^ (*n* = 107)					
BMI * (kg/m^2^) (Mean ± SD)	27.8 ± 3.1	27.1 ± 3.3	27.4 ± 3.2	26.5 ± 3.3	0.234	**0.036**	0.372	0.084
Total PCSA * (mm^2^) (Mean ± SD)	1579 ± 278	1459 ± 225	1517 ± 258	1613 ± 307	**0.015**	0.544	**0.003**	**0.035**
PMI * (mm^2^/m^2^)	618 ± 95	533 ± 85	574 ± 99	627 ± 103	**<0.001**	0.616	**<0.001**	**0.001**
PMD *	44.2 ± 4.1	43.9 ± 3.9	44.0 ± 4.0	44.4 ± 3.9	0.756	0.823	0.595	0.685
HbA1c * (%) (Mean ± SD)	7.1 ± 4.2	7.1 ± 0.5	7.1 ± 0.4	-	0.736	-	-	-
Duration of T2DM (year) (Mean ± SD)	10.3 ± 3.7	7.1 ± 2.7	8.6 ± 3.6	-	**<0.001**	-	-	-

* Independent samples *t* test, SD: Standard Deviation, BMI: Body Mass Index, PCSA: Psoas Cross-Sectional Area, PMI: Psoas Muscle Index, PMD: Psoas Muscle Density, T2DM: Type 2 Diabetes Mellitus, OAD: Oral Antidiabetics. Superscript letters were added so that *p* values could be compared in pairs.

**Table 3:** Correlation analyzes between duration of Type 2 Diabetes Mellitus (T2DM), age, psoas muscle index (PMI), and body mass index (BMI) (*r* = correlation coefficient; *p* = statistical significance).

	Insulin (*n* = 52)		OAD (*n* = 55)		Total Study Group (*n* = 107)	
	*r*	*p*	*r*	*p*	*r*	*p*
Duration of T2DM/PMI *	−0.474	**<0.001**	−0.525	**<0.001**	−0.192	**0.038**
Age/PMI **	−0.663	**<0.001**	−0.707	**<0.001**	−0.597	**<0.001**
Duration of T2DM/BMI *	0.233	0.096	0.779	**<0.001**	0.332	**<0.001**
BMI/PMI *	−0.078	0.582	−0.213	0.118	−0.093	0.338

* Pearson correlation test, ** Spearman correlation test, T2DM: Type 2 Diabetes Mellitus, PMI: Psoas Muscle Index, BMI: Body Mass Index, OAD: Oral Antidiabetic Figure Legends.

## Data Availability

The data that support the findings of this study are available from the corresponding author upon reasonable request.
